# Myeloperoxidase-Oxidized LDLs Enhance an Anti-Inflammatory M2 and Antioxidant Phenotype in Murine Macrophages

**DOI:** 10.1155/2016/8249476

**Published:** 2016-08-30

**Authors:** Valérie Pireaux, Aude Sauvage, Benoît Bihin, Martine Van Steenbrugge, Alexandre Rousseau, Pierre Van Antwerpen, Karim Zouaoui Boudjeltia, Martine Raes

**Affiliations:** ^1^URBC-Narilis, University of Namur, 61 rue de Bruxelles, 5000 Namur, Belgium; ^2^UMBD, University of Namur, 61 rue de Bruxelles, 5000 Namur, Belgium; ^3^Laboratory of Experimental Medicine (ULB 222 Unit), Université Libre de Bruxelles, CHU-Charleroi, ISPPC Hôpital Vésale, Montigny-Le-Tilleul, Belgium; ^4^Therapeutic Chemistry, ULB (Campus de la Plaine) CP205/05, boulevard du Triomphe, Brussels, Belgium

## Abstract

Macrophages and oxidized LDLs play a key role in atherogenesis but their heterogeneity has been neglected up to now. Macrophages are prone to polarization and subsets of polarized macrophages have been described in atheromas. LDLs can be oxidized not only chemically by copper (Ox-LDLs) but also enzymatically by myeloperoxidase (MpOx-LDLs) resulting in oxidized LDLs poor in lipid peroxides. The effects of physiologically relevant myeloperoxidase-oxidized LDLs on macrophage polarization or on polarized macrophages remain largely unknown. In this study, the effects of LDLs on macrophage polarization were investigated by monitoring the expression of M1 and M2 genes following stimulation with native LDLs, Ox-LDLs, or MpOx-LDLs in RAW 264.7 cells. Except for* MRC1*, which is induced only by Ox-LDLs, MpOx-LDLs induced an overexpression of most of the selected marker genes at the mRNA level. MpOx-LDLs also modulate marker gene expression in polarized macrophages favoring notably anti-inflammatory* Arg1* expression in M2 cells and also in the other phenotypes. Noteworthy, MpOx-LDLs were the most efficient to accumulate lipids intracellularly in (un)polarized macrophages whatever the phenotype. These data were largely confirmed in murine bone marrow-derived macrophages. Our data suggest that MpOx-LDLs were the most efficient to accumulate within cells and to enhance an anti-inflammatory and antioxidant phenotype in M2 cells and also in the other macrophage phenotypes.

## 1. Introduction

Cardiovascular diseases, the major cause of deaths in western societies and throughout the world, are mainly due to atherosclerosis, a chronic inflammatory disease affecting mainly medium and large arteries (WHO, fact sheets of 2016). Local blood flow perturbations or injuries lead to an increased permeability of the endothelial layer, favoring lipoprotein infiltration in the intima, where they get oxidized [1–4]. The oxidized lipoproteins are atherogenic. They activate endothelial cells increasing their chemokine (e.g., MCP-1) and cytokine (e.g., IL-6) secretion, leading to the recruitment of monocytes, which will differentiate into macrophages within the intima [3, 5–7]. However, most of the studies have focused on copper-oxidized LDLs (Ox-LDLs), while more relevant forms of oxidized LDLs have been neglected. Calay and coworkers showed that myeloperoxidase-oxidized LDLs (MpOx-LDLs) activate different signaling cascades in macrophages compared to Ox-LDLs [8]. Macrophages become foam cells following internalization of these oxidized LDLs through scavenger receptors. Noteworthy, higher intracellular accumulation levels are observed with MpOx-LDLs [8–10].

Macrophages are not homogeneous. Different signals in the cellular environment functionally activate macrophages, modulating their phenotypes as an adaptive response. Classically activated M1 or proinflammatory macrophages are mainly involved in acute host defense owing to their microbicidal activity. Exerting cytotoxic and antiproliferative activities by the production of ROS (reactive oxygen species), RNS (reactive nitrogen species), and proinflammatory cytokines (e.g., Interleukin-6), they contribute to tissue destruction and tumor resistance [11–13]. This phenotype can be induced* in vitro* by interferon-*γ* (IFN-*γ*) and/or by bacterial stimuli (e.g., LPS), and, over the last years, the combination of LPS and IFN-*γ* has become the standard for inducing “classically” polarized M1 cells in both murine and human macrophages from different sources [11, 14–19]. On the contrary, anti-inflammatory M2 macrophages resolve inflammation by the production of anti-inflammatory mediators [7, 12]. They not only are involved in matrix remodelling, angiogenesis, and tissue repair but also contribute to tumor promotion [12, 13, 20]. These alternatively activated M2 macrophages can be induced* in vitro* by Interleukin-4 (IL-4) and Interleukin-13 (IL-13) [18].

Another more recently described phenotype (MOX) is induced* in vitro* by oxidized phospholipids and characterized by high levels of expression of genes regulated by Nrf2 (nuclear factor erythroid 2-related factor 2) (e.g.,* heme oxygenase-1* and* sulfiredoxin-1*) [18, 21–23]. Although less documented, this phenotype, relevant in the context of oxidative stress and probably of atherosclerosis, has been described to represent up to 30% of the macrophages present in advanced atherosclerotic lesions, at least in LDL-R^−/−^ mice [22].

Despite many recent studies focusing on macrophage polarization, in particular in relation with several chronic diseases, the mechanisms as well as the pathophysiological significance of macrophage polarization in atheroma remain unclear.

In this study, we investigated the effect of native or modified LDLs not only on macrophage polarization but also on polarized macrophages and on foam cell formation. We compared in particular the effects of copper-oxidized and myeloperoxidase-oxidized LDLs. To do so, we first validated the use of the RAW 264.7 murine macrophage-like cell line, which is often used to produce foam cells [24, 25], as a robust and reproducible model for studying macrophage polarization into proinflammatory M1 (also named M(LPS + IFN-*γ*) according to the “Nomenclature and Experimental Guidelines” about macrophage activation and polarization [26]) and anti-inflammatory M2 (also named M(IL-4 + IL-13)) macrophages [26].

The results on RAW cells were validated using murine bone marrow-derived macrophages (BMDMs).

## 2. Materials and Methods

### 2.1. Cells and Cell Treatments

The murine RAW 264.7 macrophage cell line, obtained from the American Type Culture Collection (ATCC, Manassas, VA, USA), was grown in DHG-L1 medium (Dulbecco's modified Eagle's medium + high glucose (4,5 g/L) + NaHCO_3_ (1,5 g/L)) (SAFC Global, Lenexa, KS, USA) + 10% HIS (heat-inactivated fetal calf serum) (Gibco-Life Technologies, Carlsbad, CA, USA). Cells were seeded and treated in 6-well plates (750 000 cells/well) (Corning-Costar, Lowell, MA, USA).

RAW 264.7 cells were polarized towards M1 macrophages by adding LPS (10 ng/mL) (from* Escherichia coli* (serotype 0111:B4), obtained from Sigma-Aldrich (St. Louis, MO, USA)) in the presence of IFN-*γ* (20 ng/mL) (R&D Systems, Minneapolis, MN, USA) or towards M2 cells via the addition of IL-4 combined with IL-13 (20 ng/mL both) (R&D Systems, Minneapolis, MN, USA) for 18 h. After polarization, media were removed. Then unpolarized (M0 macrophages) and polarized macrophages were incubated for 24 h with RPMI (Ctl) (RPMI-1640 culture medium (glutamine-free) from Lonza, Basel, Switzerland), native LDLs (Nat-LDLs), CuSO_4_-oxidized LDLs (Ox-LDLs), or myeloperoxidase-oxidized LDLs (MpOx-LDLs) at 100 *μ*g/mL. RAW 264.7 macrophages were also incubated in the presence of IFN-*γ* only (20 ng/mL) as a control.

BMDMs (bone marrow-derived macrophages) were obtained from femurs and tibias of 6-to-8-week-old C57BL/6 mice. Mice were euthanized by 1-minute exposure to CO_2_ followed by cervical dislocation. Once the bone marrow was collected by flushing, cells were incubated for 7 days with DMEM (Gibco-Life Technologies, Carlsbad, CA, USA) supplemented with 10% heat-inactivated low-endotoxin serum (Sigma-Aldrich, St. Louis, MO, USA), 1% of penicillin/streptomycin (Life Technologies, Carlsbad, CA, USA), and 10% of L929 conditioned media. They were cultured in cell culture Petri dishes (Greiner Bioscience, Frickenhausen, Germany) and were seeded at a density of 500 000 cells/well in 6-well plates (Greiner Bioscience, Frickenhausen, Germany) for further analyses. Then BMDMs were polarized and/or stimulated with LDLs with the same protocol as for RAW 264.7 (protocol adapted from [27, 28]).

Mice were handled in strict accordance with good animal practice as defined by the Ethics Committee of the University of Namur. This committee approved all the work planned on animals.

Murine L-929 fibroblasts were obtained from the ATCC and incubated for 6 days with DMEM plus 10% of heat-inactivated low-endotoxin serum in order to produce conditioned medium needed to differentiate murine monocytes into macrophages (BMDMs). L929 conditioned medium is a major source of M-CSF, GM-CSF, and other factors, which stimulate the differentiation and growth of bone marrow cells into macrophages [29].

### 2.2. LDL Preparation and Oxidation

Native LDLs (Nat-LDLs) were obtained by sequential density gradient ultracentrifugation from plasma of healthy blood donors. They were prepared, oxidized, and characterized as previously described by Calay et al. [8]. The concentration of Nat-LDLs in PBS was adjusted to 1 mg/mL before incubation with 10 *μ*M copper sulfate for 24 hours at 37°C. Oxidation was stopped by the addition of 25 *μ*M butylated hydroxytoluene (Sigma-Aldrich, St. Louis, MO, USA) and incubation on ice for 1 hour. MpOx-LDLs were generated by mixing 8 *μ*L of HCl 1 M (final concentration: 4 *μ*M) (Merck, Billerica, MA, USA), 50 *μ*L of recombinant human MPO (rhMPO) 86 U/mL (final relative activity: 2.6 U/mg LDL), 1600 *μ*g of LDLs diluted in PBS, and 40 *μ*L of 50 mM H_2_O_2_ (final concentration: 1 mM) (Merck, Billerica, MA, USA). The volume was adjusted to 2 mL with PBS containing 1 g/L of EDTA (Merck, Billerica, MA, USA) at pH 6.5. rhMPO was provided by the Laboratory of Experimental Medicine (ULB 222 Unit, CHU-Charleroi, ISPPC Hôpital Vésale, Belgium). The oxidation reaction for the generation of MpOx-LDLs was carried out at 37°C for 5 minutes and stopped by incubation on ice to inhibit the MPO enzymatic activity. Nat-LDLs, Ox-LDLs, and MpOx-LDLs were desalted against RPMI-1640 without glutamine (Lonza, Belgium) by using PD-10 desalting columns (GE Healthcare, Little Chalfont, Buckinghamshire, UK). LDLs were sterile-filtered (0.2 *μ*m), stored in the dark at 4°C, and used within 4 days in order to prevent further oxidation. The LDL concentration was determined by the Lowry method and LDLs were used at a final concentration of 100 *μ*g/mL.

### 2.3. RNA Extraction and Quantitative Real-Time RT-PCR

Total RNA, from M0, M1, and M2 macrophages, was extracted according to the manufacturer's protocol using RNeasy Mini Kit (RAW 264.7) or RNeasy Micro Kit (BMDMs) (Qiagen, Hilden, GE). cDNA was synthetized from 2 *μ*g of total RNA using the Transcriptor First Strand cDNA Synthesis Kit and according to the manufacturer's instructions (Roche Applied Science, Basel, Switzerland). Forward and reverse primers for* TBP, HO-1, Srxn1, Arg1, MRC1, Mgl2, YM1, iNOS, IL-6, Arg2*, and* TNF-α* (Integrated DNA Technologies (IDT) (Coralville, IA, USA)) were designed using the Primer Express 1.5 software (Applied Biosystems, Carlsbad, CA, USA). Real-time RT-qPCR was performed using FastStart Universal SYBR Green Master (Rox) (Roche Applied Science, Basel, Switzerland). The amplification process starts with a denaturation step at 95°C for 5 minutes, followed by 40 cycles of denaturation at 95°C for 15 seconds and then annealing and extension at 65°C for 1 minute, using the 7900 HT Fast Real-Time PCR System (Applied Biosystems, Carlsbad, CA, USA). The gene encoding the Tata-Box Protein (TBP) was selected as a housekeeping gene to normalize data.

### 2.4. Flow Cytometry

M0, M1, and M2 macrophages were detached with EDTA (Merck, Billerica, MA, USA) (15 minutes at RT) and washed with PBS. After washing, cells were stained with anti-Fc monoclonal antibody (dilution 200x) (BD Pharmingen, Franklin Lakes, NJ, USA) for 20 minutes at 4°C and then incubated with primary anti-MRC1 monoclonal antibody (dilution 10x) (Abcam, Cambridge, UK) and secondary anti-IgG-Alexa-488 antibody (dilution 100x) (Invitrogen Molecular Probes, Carlsbad, CA, USA) both for 60 minutes at 4°C. Finally, they were fixed with PFA 2% (Sigma-Aldrich, St. Louis, MO, USA) for 20 minutes at 4°C. Cells incubated with only the primary or secondary antibodies were used as negative controls.

### 2.5. Western Blotting

After removing the medium, cells were washed in PBS, scraped, and lysed in 30 *μ*L of lysis buffer (10 mM Tris (Merck, Billerica, MA, USA), 100 mM NaCl (Merck, Billerica, MA, USA), 10% glycerol (Merck, Billerica, MA, USA), 1% Nonidet P-40 Substitute (Sigma-Aldrich, St. Louis, MO, USA), 0,1% sodium dodecyl sulfate (MP Biochemicals, Solon, OH, USA), 0,5% sodium deoxycholate (Merck, Billerica, MA, USA)) containing a proteinase inhibitor “Complete” cocktail (Roche Molecular Biochemicals, Basel, Switzerland) and phosphatase inhibitors (25 mM Na_3_VO_4_, 250 mM 4-nitrophenyl phosphate (Sigma-Aldrich, St. Louis, MO, USA), 250 mM *β*-glycerophosphate (VWR, Radnor, PA, USA), and 125 mM NaF (Merck, Billerica, MA, USA) at a 1/25 dilution in H_2_O) for 30 minutes at 4°C. Lysates were then centrifuged at 15 700 rcf for 10 minutes (Eppendorf Microcentrifuge 5415R). Protein concentration was determined using the Pierce 660 protein assay and 15 *μ*g (RAW 264.7 cells) or 5 *μ*g (BMDMs) of proteins was loaded on 4–20% SDS-PAGE gels (Bio-Rad Laboratories, Hercules, CA, USA). Proteins were transferred onto a polyvinylidene fluoride (PVDF) membrane (0,45 *μ*m) (Millipore, Billerica, MA, USA) for 2 hours at 70 V. Primary antibodies are mouse anti-HO-1 (heme oxygenase-1) monoclonal antibody (Thermo Scientific, Waltham, MA, USA), rabbit anti-sulfiredoxin polyclonal antibody (Proteintech, Chicago, IL, USA), and rabbit anti-TBP polyclonal antibody (Santa Cruz Biotechnology, Dallas, TX, USA) (dilution 1000x). Secondary antibodies are either IRDye 800CW goat anti-mouse antibodies or IRDye 680 goat anti-rabbit antibodies (LI-COR, Lincoln, NE, USA) (dilution 10000x). Quantitative analysis of fluorescence intensity was performed using the Odyssey Classic Infrared Imaging System (LI-COR, Lincoln, NE, USA).

### 2.6. ELISA Assay

Supernatants were harvested. They were then centrifuged at 15 700 rcf at 4°C and pellets were discarded. Mouse IL-6, IL-12, TNF-*α*, and IL-10 ELISA assays were performed following manufacturer's instructions (R&D Systems, Minneapolis, MN, USA).

### 2.7. Analysis of the Number and Size of Lipid Droplets

Cells seeded in 96-well plates were fixed with PFA 4% for 10 minutes at RT. After washing with PBS, they were permeabilized with PBS-Triton 1% for 5 minutes at RT, washed with PBS-BSA 2%, and incubated with BODIPY 493/503 (dilution: 1/40) (Invitrogen Molecular Probes, Carlsbad, CA, USA) for 2 hours in the dark at RT. They were finally incubated with Phalloidin-Alexa 555 (dilution: 1/50) (Invitrogen Molecular Probes, Carlsbad, CA, USA) and Hoechst FluoroPure grade (dilution: 1/1000) (Invitrogen Molecular Probes, Carlsbad, CA, USA) for 30 minutes in the dark at RT and analyzed using the BD Pathway 855, with the AttoVision software and BD-IDE software (lens: 20x) (Becton Dickinson, Franklin Lakes, NJ, USA).

### 2.8. Cholesterol Quantitation

Cells were seeded in 6-well plates at a density of 1 · 10^6  ^cells/well, polarized for 18 hours, and incubated or not with LDLs for 24 hours. After this incubation, cholesterol was extracted from cells by adding 200 *μ*L of chloroform : isopropanol : IGEPAL CA-630 (7 : 11 : 0.1) in a microhomogenizer. The solution was then centrifuged at 13 000 ×g for 10 minutes in order to remove insoluble material. The organic phase was transferred in a new tube, air-dried at 50°C to remove chloroform, and put under vacuum for 30 minutes to remove any residual organic solvent. The dried lipids were dissolved with 220 *μ*L of the cholesterol assay buffer and were vortexed until the lipid solution was homogeneous. Cholesterol was finally quantified following the manufacturer's instructions by using reaction mixes containing the cholesterol assay buffer, probe, enzyme mix, and/or cholesterol esterase. The absorbance was measured at 570 nm (Cholesterol Quantitation Kit MAK043) (Sigma-Aldrich, St. Louis, MO, USA) and results were expressed as *μ*g/*μ*L of free, esterified, or total cholesterol following the manufacturer's instructions.

### 2.9. Phagocytosis Assay

Cells were seeded in 12-well plates at a density of 5 · 10^5^ cells/well and were polarized for 18 hours. They were then incubated or not with LDLs (100 *μ*g/mL) and fluorescent beads (dilution: 1/133) for 24 hours. Phagocytosis of fluorescent beads was measured by flow cytometry, using the FACS BD Verse (Becton Dickinson, Franklin Lakes, NJ, USA). The phagocytosis assay of fluorescent beads was performed following manufacturer's instructions (Phagocytosis Assay Kit (IgG FITC) Item number 500290) (Cayman Chemical, Ann Arbor, MI, USA).

### 2.10. Statistical Analysis

R (version 3.0.3; the R Foundation for Statistical Computing) was used for statistical analysis. As RT-qPCR data presents highly heterogeneous variances, statistical analyses were performed on log-transformed data and, in order to facilitate interpretation, untransformed data are shown (Figures 4(a), 4(b2), 5, and 7(b), and S1C, and S1D in Supplementary Material available online at http://dx.doi.org/10.1155/2016/8249476).

Before measuring the mean comparisons, homoscedasticity was assessed with Bartlett's test.

**Figure 1:**
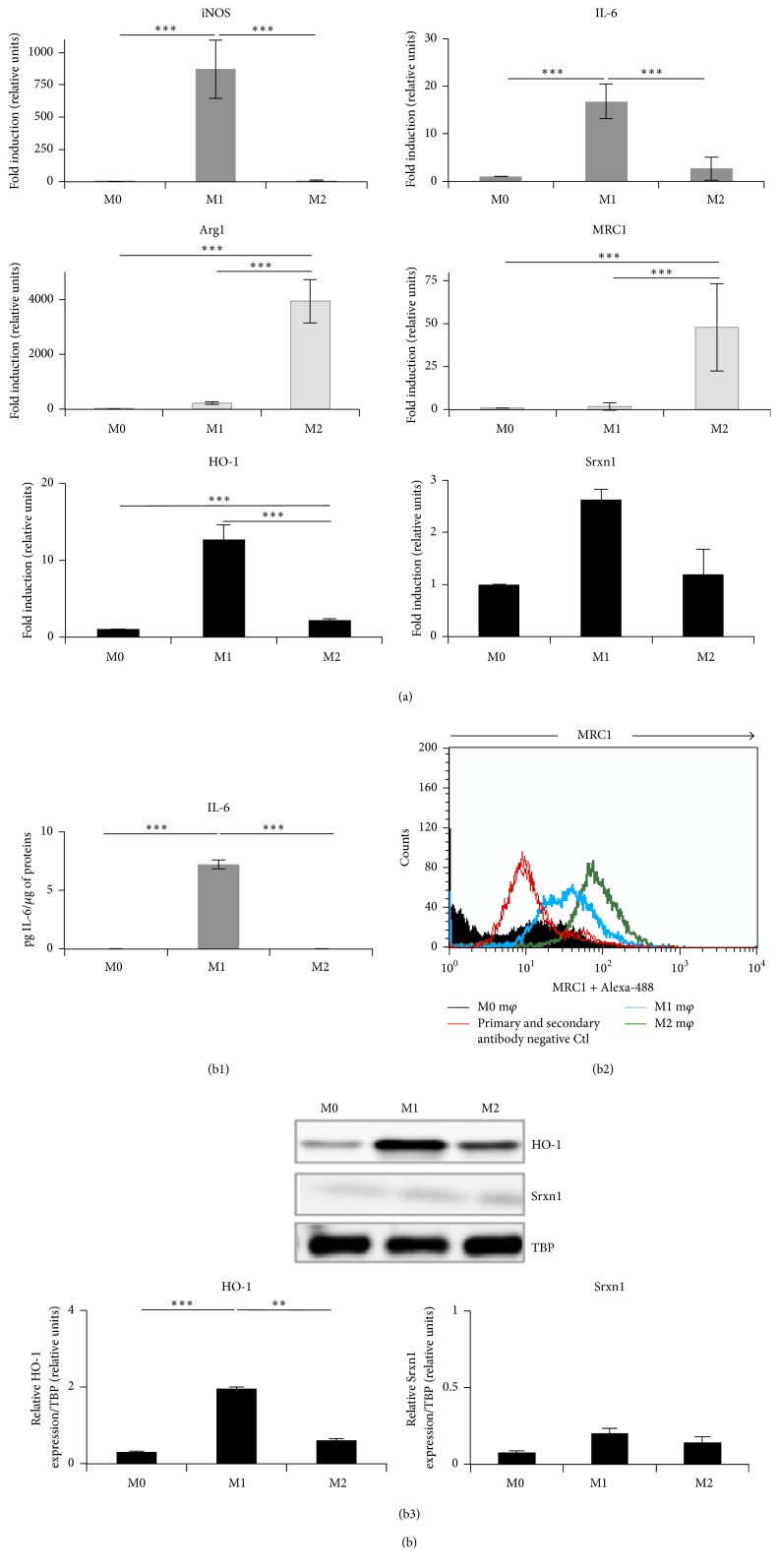
Characterization of RAW 264.7 M0, M1 (LPS + IFN-*γ*), and M2 (IL-4 + IL-13) polarized macrophages. Cells were polarized as described in Materials and Methods. (a) Expression of polarization marker genes at the mRNA level (RT-qPCR), in M0, M1, and M2 macrophages. The expression of* iNOS* and* IL-6 *as M1 markers,* Arg1* and* MRC1 *as M2 markers, and* HO-1* and* Srxn1 *as MOX redox-sensitive markers was analyzed by RT-qPCR. Data were normalized with* TBP* used as housekeeping gene and expressed as mean fold induction relatively to M0 cells ± SD (*n* = 3). (b) Expression of polarization markers at the protein level in M0, M1, and M2 macrophages. (b1) Production of secreted IL-6 (M1 marker) in cell culture supernatants assessed by ELISA. Data are expressed relatively per *μ*g of protein per well as mean ± SD (*n* = 3). (b2) Surface expression of MRC1 (M2 marker) analyzed by flow cytometry in M0, M1, and M2 macrophages (m*φ*). GMF (geometric mean of the fluorescence intensity) values are reported in arbitrary units: ΔGMF_M2-M0_, 65.57 and ΔGMF_M1-M0_, 28.9. The data presented are representative of 3 independent experiments. (b3) Expression of HO-1 and Srxn1 assessed by Western blotting in M0, M1, and M2 macrophages. Data are normalized with TBP used as loading control and expressed as mean ± SD (*n* = 3). Results are representative of 3 independent experiments. ANOVA 1: ^*∗∗*^ *p* < 0.01 and ^*∗∗∗*^ *p* < 0.001.

**Figure 2:**
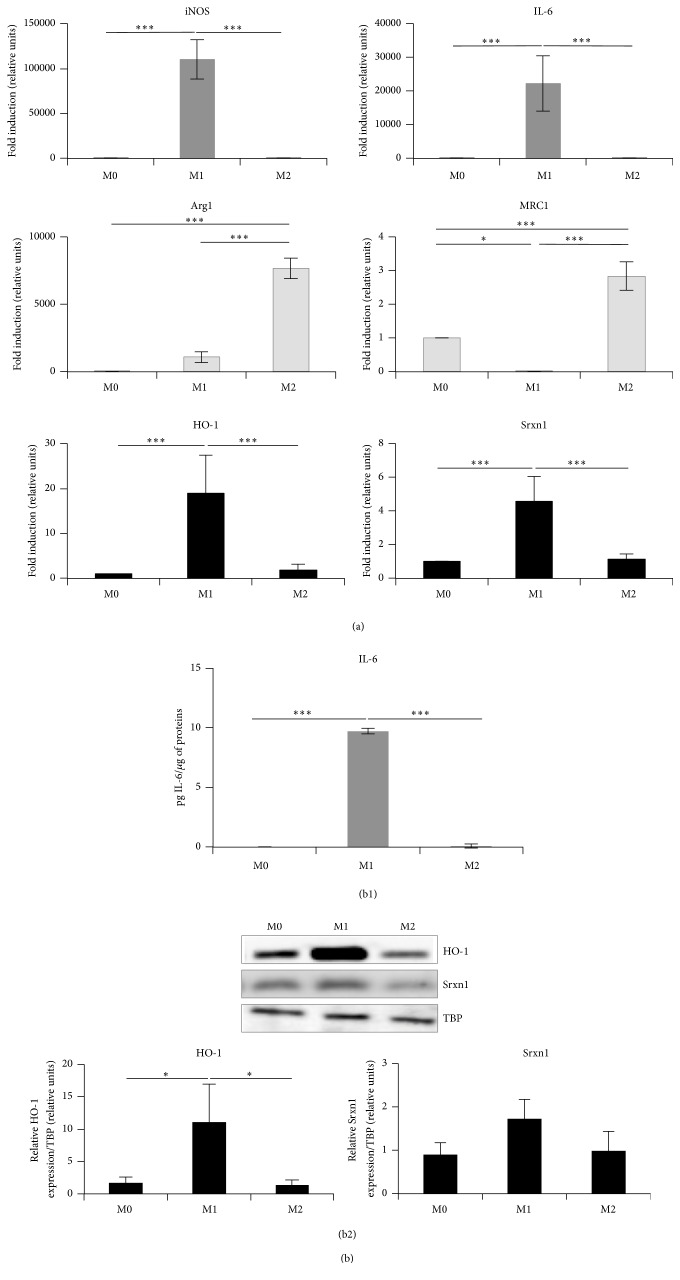
Characterization of M0, M1, and M2 polarized BMDMs. (a) Expression of polarization marker genes at the mRNA level (RT-qPCR) in M0, M1, and M2 macrophages. The expression of marker genes was analyzed as described in Figure 1 and the data were expressed as mean fold induction relatively to M0 cells ± SD (*n* = 3). (b) Expression of polarization markers at the protein level in M0, M1, and M2 macrophages. (b1) Production of secreted IL-6 (M1 marker) in cell culture supernatants assessed by ELISA. Data are expressed relatively per *μ*g of protein per well as mean ± SD (*n* = 3). (b2) Expression of HO-1 and Srxn1 assessed by Western blotting in M0, M1, and M2 macrophages. Data are normalized with TBP used as loading control and expressed as mean ± SD (*n* = 4). Results are representative of 4 independent experiments. ANOVA 1: ^*∗*^ *p* < 0.05 and ^*∗∗∗*^ *p* < 0.001.

**Figure 3:**
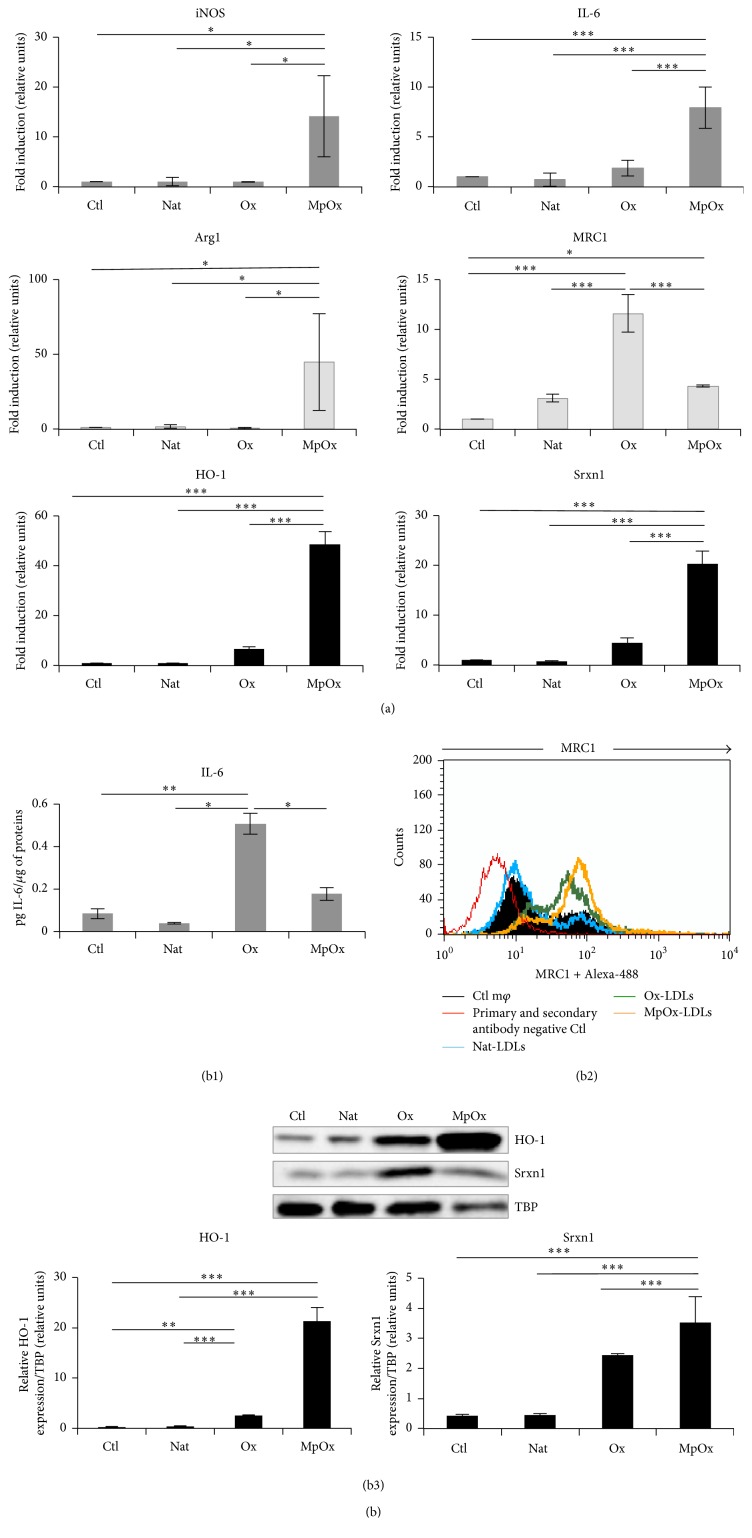
Comparative effects of LDLs on marker gene expression in unpolarized RAW 264.7 M0 macrophages. M0 macrophages were treated for 24 hours in the presence of medium alone (Ctl), native LDLs (Nat), Ox-LDLs (Ox), or MpOx-LDLs (MpOx) (100 *μ*g/mL). (a) Expression of polarization marker genes at the mRNA level (RT-qPCR). The expression of marker genes was analyzed by RT-qPCR as in Figure 1. Data are normalized with TBP used as housekeeping gene and expressed as mean fold induction relatively to Ctl cells ± SD (*n* = 3). (b) Expression of polarization markers at the protein level (FACS, ELISA, and WB) in Ctl and Nat-LDLs-, Ox-LDLs-, and MpOx-LDLs-treated M0 macrophages. (b1). Production of secreted IL-6 (M1 marker) in cell culture supernatants assessed by ELISA. Data are expressed relatively per *μ*g of protein per well as mean ± SD (*n* = 3). (b2) Surface expression of MRC1 (M2 marker) analyzed by flow cytometry in Ctl and treated macrophages (m*φ*). GMF values are reported in arbitrary units: ΔGMF_Nat-CTL_, 0; ΔGMF_Ox-CTL_, 20.75; and ΔGMF_MpOx-CTL_, 33.82. The data presented are representative of 3 independent experiments. (b3) Expression of HO-1 and Srxn1 assessed by Western blotting in Ctl and Nat-LDLs-, Ox-LDLs-, and MpOx-LDLs-treated macrophages. Data are normalized with TBP used as loading control and expressed as mean ± SD (*n* = 3). Results are representative of 3 independent experiments. ANOVA 1: ^*∗*^ *p* < 0.05; ^*∗∗*^ *p* < 0.01; and ^*∗∗∗*^ *p* < 0.001.

**Figure 4:**
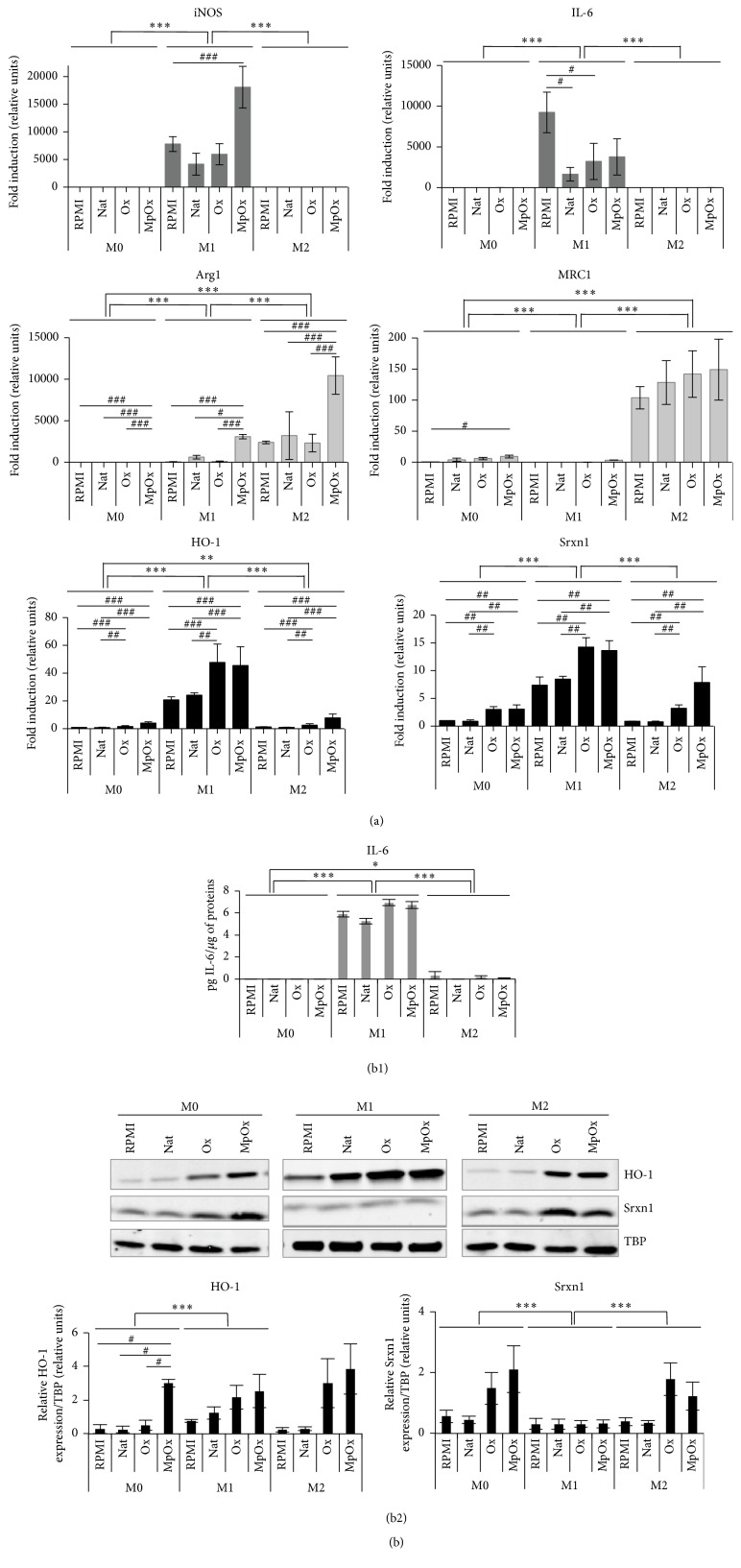
Comparative effects of LDLs on (un)polarized RAW 264.7 macrophages. M0, M1, and M2 macrophages were stimulated in the presence or absence (RPMI control) of Nat-LDLs, Ox-LDLs, and MpOx-LDLs for 24 hours (100 *μ*g/mL). (a) Expression of polarization marker genes at the mRNA level (RT-qPCR). The expression of marker genes was analyzed by RT-qPCR as described in Figure 1. Data are normalized with TBP used as housekeeping gene and expressed as mean fold induction relatively to M0 cells in RPMI ± SD (*n* = 6). Data for* MRC1* were analyzed by a Kruskal-Wallis ANOVA on ranks. Zoomed data for* Arg1* and* MRC1* are presented in Figure S5. (b). Expression of polarization marker genes at the protein level (ELISA; WB). (b1) Production of secreted IL-6 (M1 marker) in cell culture supernatants assessed by ELISA. Data are expressed relatively per *μ*g of protein per well as mean ± SD (*n* = 3). (b2) Expression of HO-1 and Srxn1 assessed by Western blotting. Data are normalized with TBP used as loading control and expressed as mean ± SD (*n* = 3). Results are representative of 3 independent experiments. ANOVA 2: ^*∗*, #^ *p* < 0.05; ^*∗∗*, ##^ *p* < 0.01; and ^*∗∗∗*, ###^ *p* < 0.001.

**Figure 5:**
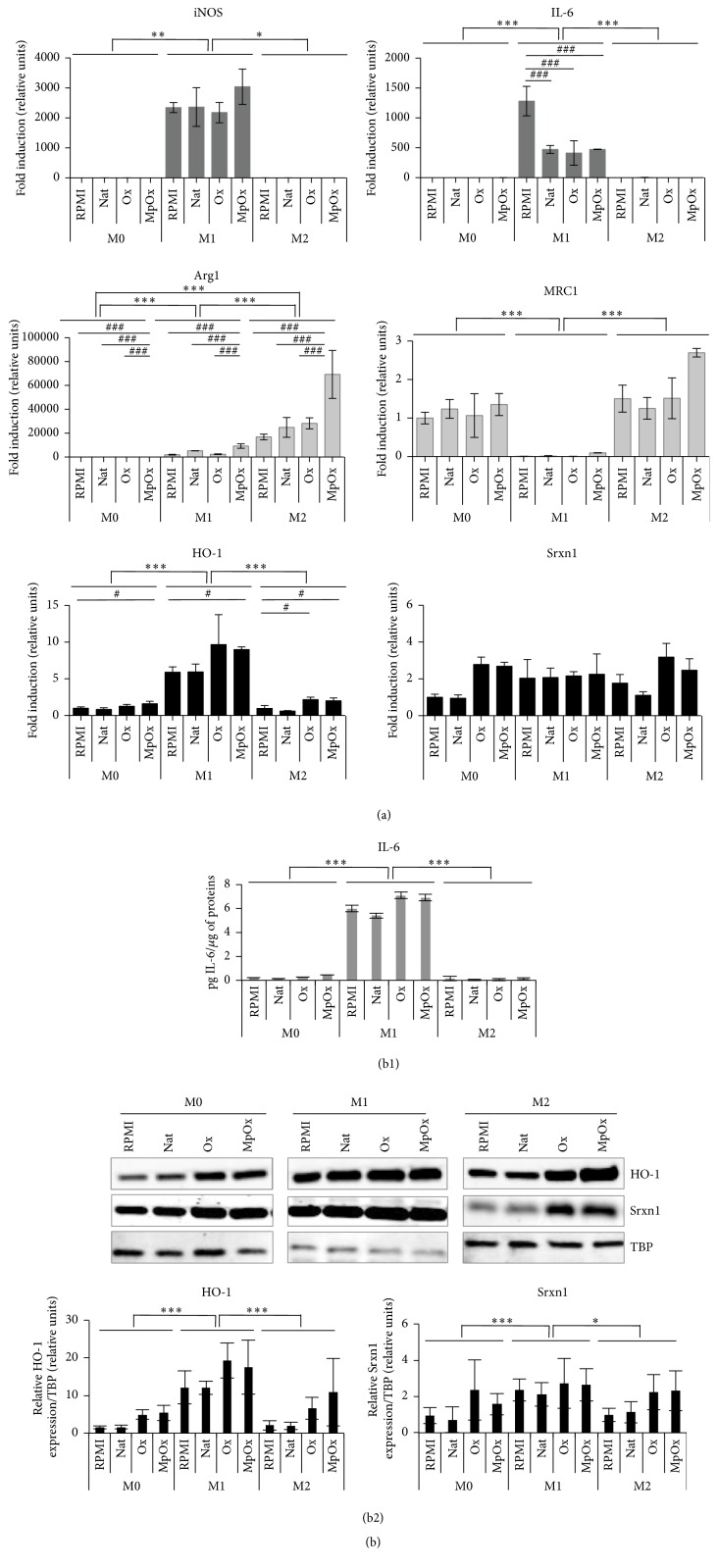
Comparative effects of LDLs on (un)polarized BMDMs. M0, M1, and M2 macrophages were stimulated in the presence or absence (RPMI control) of Nat-LDLs, Ox-LDLs, and MpOx-LDLs for 24 hours (100 *μ*g/mL). (a) Expression of polarization marker genes at the mRNA level (RT-qPCR). The expression of marker genes was analyzed by RT-qPCR as described in the preceding figures. Data are normalized with TBP used as housekeeping gene and expressed as mean fold induction relatively to M0 cells in RPMI ± SD (*n* = 5). Data were analyzed by a Kruskal-Wallis ANOVA on ranks (except for* Arg1* and* IL-6*: two-way ANOVA test). (b) Expression of polarization markers at the protein level (ELISA; WB). (b1) Production of secreted IL-6 (M1 marker) in cell culture supernatants assessed by ELISA. Data are expressed relatively per *μ*g of protein per well as mean ± SD (*n* = 3). (b2) Expression of HO-1 and Srxn1 assessed by Western blotting. Data are normalized with TBP used as loading control and expressed as mean ± SD (*n* = 4). Results are representative of 4 independent experiments. ANOVA 2: ^*∗*, #^ *p* < 0.05; ^*∗∗*^ *p* < 0.01; and ^*∗∗∗*, ###^ *p* < 0.001."

**Figure 6:**
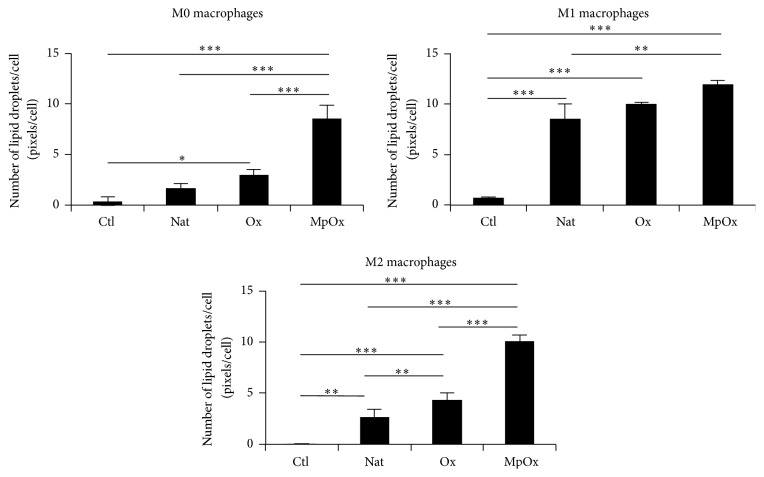
Comparative effects of LDLs on (un)polarized RAW 264.7 macrophages to induce foam cells. M0, M1, and M2 macrophages were incubated in the presence or absence of Nat-LDLs, Ox-LDLs, and MpOx-LDLs (100 *μ*g/mL) for 24 hours. The number of lipid droplets was estimated using a High Content Imager, as described in Materials and Methods. Data are expressed as mean ± SD (*n* = 3). ANOVA 1: ^*∗*^ *p* < 0.05; ^*∗∗*^ *p* < 0.01; and ^*∗∗∗*^ *p* < 0.001.

**Figure 7:**
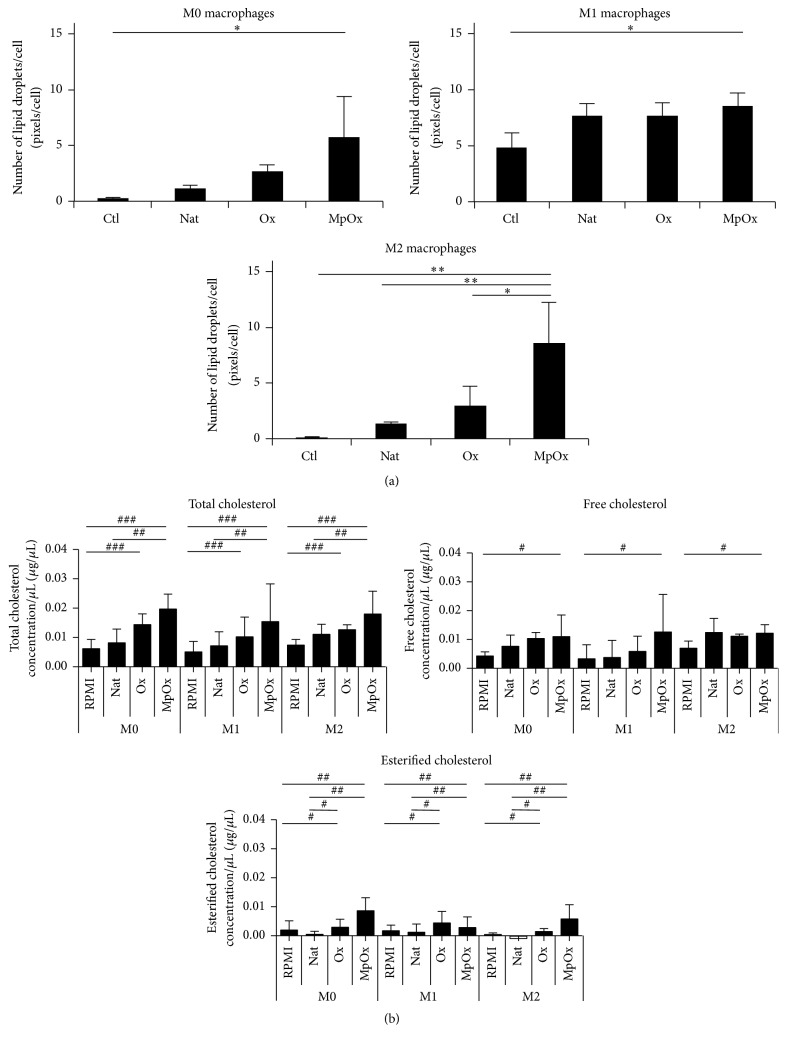
Comparative effects of LDLs on (un)polarized BMDMs to induce foam cells. (a) M0, M1, and M2 macrophages were incubated in the presence or absence of Nat-LDLs, Ox-LDLs, and MpOx-LDLs (100 *μ*g/mL) for 24 hours. The number of lipid droplets was estimated using a High Content Imager, as described in Materials and Methods. Data are expressed as mean ± SD (*n* = 4). ANOVA 1: ^*∗*^ *p* < 0.05 and ^*∗∗*^ *p* < 0.01. (b) Intracellular cholesterol content in M0, M1, and M2 macrophages incubated in the presence or absence of Nat-LDLs, Ox-LDLs, and MpOx-LDLs for 24 hours. The intracellular cholesterol was measured by a colorimetric assay (absorbance: 570 nm). Data are expressed as *μ*g/*μ*L of free, esterified, and total cholesterol (mean ± SD (*n* = 3)). Data were analyzed by a Kruskal-Wallis ANOVA on ranks. ANOVA 1: ^*∗*^ *p* < 0.05 and ^*∗∗*^ *p* < 0.01.

When homoscedasticity could be assumed (*p value *> 0.05), we performed one-way (Figures 1, 2, 3, 6, 7(a), S1A, S1B, S2A, S3A, and S4) or two-way ANOVA according to the experimental design.* Post hoc* pairwise mean comparisons were then performed with Tukey's method. Following two-way ANOVA, pairwise comparisons were performed among marginal means if there was no statistically significant interaction and among cell means otherwise (Figures 4(a) and S5: except* MRC1*, Figures 4(b) and 5(a):* Arg1* and* IL-6*, and Figures 5(b) and S6).

When homoscedasticity could not be assumed (*p value *< 0.05), we performed Kruskal-Wallis rank-sum test followed by pairwise comparisons on main effects using Wilcoxon rank-sum test (Figures 4(a) and S5:* MRC1*, Figure 5(a): except for* Arg1 *and* IL-6*, and Figures 7(b), S1C, S1D, S2B, and S3B).

Data were expressed as mean ± standard error of the mean.

## 3. Results

### 3.1. RAW 264.7 Macrophages Can Be Polarized into M1 and M2 Macrophages

Unpolarized RAW 264.7 macrophages (M0) were polarized into M1 and M2 macrophages using LPS and IFN-*γ* for the M1 phenotype and IL-4 and IL-13 for the M2 phenotype [14, 19, 22]. We confirmed the respective phenotypes by measuring the expression of marker genes [30–33] both at the mRNA level and at the protein level (Figures 1, S1A, S2A, and S3A (see Supplementary materials)). At the mRNA level, M1 macrophages overexpressed proinflammatory genes, such as* inducible Nitric Oxide Synthase* and* Interleukin-6* (Figure 1(a)) as well as* Arg2* and* TNF-α* (Figure S2A), while M0 and M2 macrophages did not. Contrary to M0 and M1 macrophages, M2 macrophages overexpressed anti-inflammatory genes, such as* arginase-1* and* mannose receptor-C1 (MRC1*) (Figure 1(a)) as well as* Mgl2* (macrophage galactose N-acetyl-galactosamine specific lectin 2) and* YM1* (beta-N-acetylhexosaminidase, also known as Chil3, chitinase-like protein 3) (Figure S1A). In M1 cells, we also observed an overexpression of* heme oxygenase-1 *(*HO-1*) and* sulfiredoxin-1 *(*Srxn1*). The latter are driven by* Nrf2* and considered as markers of oxidative stress as well as of the so-called MOX macrophages, a less documented phenotype [22] (Figure 1(a)). Increased expression of some marker genes was confirmed at the protein level, respectively, by ELISA, for secreted IL-6 (Figure 1(b1)), and by flow cytometry analysis, for MRC1 (Figure 1(b2)). We also checked, by ELISA, the expression of IL-10, generally considered as an anti-inflammatory cytokine (M2 marker), which was higher in M2 macrophages, and the expression of both proinflammatory cytokines IL-12 and TNF-*α* (M1 markers), which was higher in M1 macrophages (Figure S3A). Western blot analysis confirmed that HO-1 abundance was higher in M1 macrophages, while no clear difference in abundance was observed for Srxn1 (Figure 1(b3)). IFN-*γ* alone induced to some extent the secretion of TNF-*α* but was unable to polarize the cells into M1 cells (data not shown).

The same protocols of polarization were applied to bone marrow-derived macrophages (BMDMs) obtained from the differentiation of bone marrow cells collected from femurs and tibias of 6-to-8-week-old C57BL/6 mice (Figures 2 and S1B). We observed the same trend when considering the expression of M1 and M2 markers (especially* arginase-1, Mgl2, *and* YM1*) in M1 and M2 macrophages, respectively (Figures 2(a) and S1B). The redox-sensitive genes,* HO-1* and* Srxn1*, were also overexpressed in M1 macrophages.

In summary, these results show that the RAW 264.7 cells can easily be polarized into M1 or M2 macrophages, displaying marker gene expression patterns consistent with those observed in polarized BMDMs. Fold inductions are generally higher in BMDMs and especially in M1 BMDMs (Figure 2(a)).

### 3.2. Myeloperoxidase-Oxidized LDLs Are the Most Efficient in Modulating Polarization Gene Marker Expression

Using the validated RAW 264.7 macrophage model, we evaluated the potential of LDLs to polarize macrophages. Unpolarized macrophages were stimulated with native LDLs (Nat-LDLs), copper sulfate-oxidized LDLs (Ox-LDLs), and myeloperoxidase-oxidized LDLs (MpOx-LDLs) and the expression of M1 and M2 marker genes was determined at the mRNA (Figure 3(a)) and protein levels (Figures 3(b)). While copper sulfate oxidation alters both the protein and lipid moieties, myeloperoxidase oxidizes mainly Apo-B100, generating low levels of lipid hydroperoxides [34–36].

First, we assessed the mRNA abundance of the selected marker genes and observed that native LDLs have no marked effect on macrophage polarization. Ox-LDLs mainly induced an increased expression of* MRC1*, a M2 marker gene (*p* < 0.001) [32]. MpOx-LDLs were the most potent to induce the expression of all the selected marker genes (*iNOS*,* Arg1*, and* MRC1*: *p* < 0.05;* IL-6*,* HO-1*, and* Srxn1*: *p* < 0.001) (Figure 3(a)).

While the M1 markers* iNOS* and* IL-6* and the M2 marker* Arg1* were only induced following MpOx-LDLs treatment, both Ox-LDLs- and MpOx-LDLs-treated cells overexpressed the M2 marker* MRC1* (Figure 3(a)). However, the fold induction at the mRNA level for* iNOS*,* Arg1,* and* MRC1* remained significantly lower compared to polarized M1 and M2 macrophages (Figures 1(a) and 3(a)). At the protein level (Figure 3(b2)), MRC1 abundance was higher in the presence of both Ox-LDLs and MpOx-LDLs, but in the presence of MpOx-LDLs a majority of cells clearly expressed higher levels of MRC1, compared to Ox-LDLs-treated cells. The effect of LDLs was also tested on the secretion of various cytokines. In contrast to the mRNA data, IL-6 abundance was higher in the presence of Ox-LDLs (*p* < 0.001) (Figure 3(b1)), while no significant effect was observed for IL-10, IL-12, and TNF-*α* whatever the LDLs used as compared to Ctl cells (Figure S4). HO-1 and Srxn1 expressions increased at the protein level in unpolarized cells treated with both Ox-LDLs (HO-1: *p* < 0.01) and MpOx-LDLs (HO-1 and Srxn1: *p* < 0.001) but more effectively with the latter (Figure 3(b3)). Overall, these data suggest that MpOx-LDLs by themselves tend to favor an intermediate phenotype slightly inducing the expression of some M2 markers and antioxidant enzymes.

### 3.3. Ox-LDLs and MpOx-LDLs Differentially Modulate the Phenotype of Polarized Macrophages

We next investigated the possible influence of LDLs on polarized macrophages. M1 and M2 RAW 264.7 cells (Figures 4, S1C, S2B, S3B, and S5) and BMDMs (Figures 5 and S1D) were stimulated with native LDLs, Ox-LDLs, and MpOx-LDLs. Incubation in RPMI medium devoid of LDLs was also performed and the level of expression of the polarization markers in M0 RPMI incubated cells was used as the reference condition.

First, our data show that, in particular for M1 and M2 cells, marker gene overexpression is maintained compared to M0 cells whatever the treatment. However, LDLs, and in particular MpOx-LDLs, seem to exert some modulatory effects. As there is no interaction between variables, we were able to look at the main effects for M1 and for M2 cells. Regarding the proinflammatory markers, we can conclude that* iNOS* expression in M1 cells seemed to be affected by MpOx-LDLs, especially in RAW 264.7 cells, but not by Ox-LDLs (RAW 264.7: MpOx-LDLs versus RPMI *p* < 0.001, Ox-LDLs versus RPMI *p* > 0.05; BMDMs: *p* > 0.05), while* IL-6* mRNA abundance was in general decreased in the presence of LDLs (RAW 264.7: Nat-LDLs versus RPMI *p* < 0.05, Ox-LDLs versus RPMI *p* < 0.05, and MpOx-LDLs versus RPMI *p* > 0.05; BMDMs: Nat-LDLs versus RPMI *p* < 0.001, Ox-LDLs versus RPMI *p* < 0.001, and MpOx-LDLs versus RPMI *p* < 0.001) (Figures 4(a) and 5(a)), although this difference was not confirmed at the protein level (Figures 4(b) and 5(b)). Interestingly, we observed the same effects, as for* IL-6*, with* TNF-α* at the mRNA level (Nat-LDLs versus RPMI *p* < 0.05, Ox-LDLs versus RPMI *p* < 0.05, and MpOx-LDLs versus RPMI *p* < 0.01) (Figure S2B). The mRNA expression of* Arg2* was maintained in M1 macrophages whatever the treatment (native or modified LDLs versus RPMI *p* > 0.05) (Figure S2B). At the protein level, the abundance of IL-6, IL-12, and TNF-*α* was maintained in M1 RAW 264.7 cells whatever the treatment (*p* > 0.05) (Figures 4(b1) and S3B). As well as for M1 cells, we looked for the main effects on M2 cells. The expression of* Arg1*, an anti-inflammatory gene and M2 marker, appears to be reinforced both in RAW 264.7 (Figure 4(a)) and in bone marrow-derived (Figure 5(a)) M2 macrophages incubated with MpOx-LDLs (*p* < 0.001) but not in the presence of Ox-LDLs (*p* > 0.05) (Figures 4(a) and 5(a)). When compared to results of* Arg1* expression, the effects of MpOx-LDLs on* MRC1* expression in M2 cells were similar in BMDMs but were less clear in RAW 264.7 cells (*p* > 0.05). Furthermore, when zooming Figure 4(a) (in Figure S5), we observed that MpOx-LDLs also slightly induced* Arg1* (*p* < 0.001) and* MRC1 *(*p* > 0.05, with similar patterns between replicates; except in M0: *p* < 0.05) in M0 and M1 macrophages.

At the protein level (Figure S3B), we also observed that MpOx-LDLs induce an increased secretion of IL-10 in M0 cells (MpOx-LDLs versus RPMI *p* < 0.01). In M1 cells, there seems to be a similar trend, but the effect is not significant (*p* > 0.05) probably because of the prevalent proinflammatory effect of LPS. Interestingly, in IFN-*γ*-treated macrophages, we also observed a significant induction of IL-10 (data not shown), confirming the anti-inflammatory properties of MpOx-LDLs. The expression of IL-10 was maintained in M2 macrophages whatever the treatment (*p* > 0.05). There were no major noticeable effects for IL-12 or TNF-*α* whatever the treatment.

In bone marrow-derived macrophages, it has to be mentioned that M0 macrophages, possibly due to the presence of M-CSF in the differentiation medium, expressed MRC1 at both the mRNA level (Figure 5(a)) and at protein level (data not shown) [29, 37, 38]. By looking at the main effects for the following conditions because there is no interaction between variables, we can conclude that* MRC1* expression is low in M1 cells (RAW 264.7: *p* < 0.001; BMDMs: *p* < 0.001) but is maintained in M2 cells (RAW 264.7: *p* < 0.001; BMDMs: *p* > 0.05) with no significant effect of any kind of LDLs (Figure 5(a)).

The expression of two other M2 markers,* Mgl2* and* YM1*, was evaluated in M0, M1, and M2 macrophages incubated with oxidized LDLs or not (Figures S1C and S1D). However, only* YM1* expression in RAW 264.7 cells was increased by MpOx-LDLs whatever the phenotype (*p* < 0.05 in M1 and M2 cells) (Figure S1C). The effects of MpOx-LDLs on* YM1* expression were less clear in BMDMs (Figure S1D).

Regarding the* Nrf2* driven genes, so-called MOX gene markers, our data clearly show that they are less specific. Comparing the main effects between the different phenotypes, we can come to the conclusion that M1 cells (RAW 264.7 and BMDMs) clearly strongly overexpress* HO-1* (RAW 264.7: *p* < 0.001; BMDMs: *p* < 0.001), probably due to the presence of LPS [39–41]. This overexpression is, however, reinforced in the presence of oxidized LDLs (in those conditions, RAW 264.7: *p* < 0.001; BMDMs: Ox-LDLs versus RPMI *p* > 0.05, MpOx-LDLs versus RPMI *p* < 0.05). It has to be mentioned that we also observe a slight induction of* HO-1* in M0 and M2 cells treated with oxidized LDLs, known to activate Nrf2, compared to RPMI medium and native LDLs (in M0 and M2 cells treated either with Ox-LDLs (RAW 264.7 cells: *p* < 0.001; BMDMs: *p* > 0.05 (M0), *p* < 0.05 (M2)) or with MpOx-LDLs (RAW 264.7: *p* < 0.001; BMDMs: *p* < 0.05)), which was confirmed at the protein level (Figure 4(b2) for RAW 264.7 cells and Figure 5(b2) for BMDMs).

We observed partially the same trend for* Srxn1* at least in RAW 264.7 polarized macrophages at the mRNA level (*p* < 0.01) (Figure 4(a)). In (un)polarized BMDMs, the effects of (un)modified LDLs on* Srxn1* expression were less clear (Figures 5(a) and 5(b2)).

Finally, we wanted to check to what extent LDLs were able to modulate the phagocytosis potential of (un)polarized macrophages (Figure S6). M1 cells seemed slightly more efficient (nonsignificant) to internalize beads, which is in agreement with their functions. It has to be mentioned that Fc*γ*R3, one of the potential receptors for the IgG coated latex beads, is overexpressed in M1 macrophages, compared to M0 and M2 RAW 264.7 macrophages (data not shown) [42, 43]. However, we were unable to demonstrate any effect of LDLs on this process whatever the macrophage source or phenotype. A similar trend, although less pronounced, was observed in BMDMs (Figure S6B).

### 3.4. Comparative Effects of (Un)Modified LDLs on the Differentiation of Polarized Macrophages into Foam Cells

Macrophage differentiation into foam cells was evaluated by measuring the number and the size of these lipid droplets per cell in RAW 264.7 cells (Figure 6) and in BMDMs (Figure 7(a)). MpOx-LDLs were the most efficient to induce lipid accumulation in M0 and M2 macrophages when compared to native LDLs and Ox-LDLs. M1 cells, contrary to the other phenotypes, engulfed not only oxidized but also native LDLs, probably due to their oxidative metabolism. The mean surface areas of lipid droplets within these cells were consistent with the data presented in Figures 6 and 7(a) (data not shown). Similar results were obtained on RAW 264.7 cells (Figure 6) and on BMDMs (Figure 7(a)).

We also evaluated the intracellular cholesterol content. In BMDMs (Figure 7(b)), the intracellular content of cholesterol increased in the presence of oxidized LDLs, which is in agreement with the data on lipid droplets (Figure 7(a)), with MpOx-LDLs being the most efficient to contribute to the increase of intracellular cholesterol.

However, in RAW 264.7 macrophages, no significant differences were observed. This discrepancy could be explained by a high basal content of cholesterol (8-fold compared to BMDMs). Because of this high basal content, small variations in intracellular cholesterol, after endocytosis, would be difficult to assess due to a lack of sensitivity of the assay (data not shown).

## 4. Discussion

In the present study, we showed that RAW 264.7 macrophages, commonly used as a model in studies related to atherosclerosis and prone to differentiate into foam cells [8, 44–46], can be polarized towards M1 and M2 phenotypes. Furthermore, we showed that MpOx-LDLs were the most efficient to interfere with macrophage polarization and enhance an anti-inflammatory and antioxidant phenotype.

Macrophages, and more specifically foam cells, are considered as key players in the initiation and the evolution of atherosclerotic lesions, by building up the lesion and taking part in the amplification of the inflammatory response (e.g., production of proinflammatory cytokines and growth factors) [5, 47]. However, macrophages are heterogeneous and it is only recently that the polarization of macrophages has been considered in this context not only in murine models [22, 30] but also in human lesions [48, 49]. The significance of macrophage polarization in atherogenesis and the identity of the major microenvironmental factors present in the lesion that drive macrophage polarization, as the lesion evolves, remain, however, largely unknown [7, 49–51].

Macrophage polarization has been mainly characterized* in vitro* using different models. Khallou-Laschet and coworkers obtained polarized macrophages from bone marrow-derived cells of 6-to-10-week-old C57BL/6 or ApoE^−/−^ mice [30]. Lopez-Castejón et al. polarized peritoneal macrophages isolated from C57BL/6 mice [31, 52]. Human circulating monocytes isolated from healthy human donors and differentiated into macrophages by adding M-CSF to the culture medium were polarized into M1 and M2 cells [32, 53]. Human macrophages differentiated from monocytic THP-1 cells (see, for instance, [54]) or derived from induced pluripotent stem cells have also been polarized [19].

Macrophage activation and polarization are complex phenomena giving rise to confusing descriptors in the literature. Recently, a group of macrophage biologists proposed “Nomenclature and Experimental Guidelines” for a consensus macrophage activation/polarization nomenclature. In this paper, we maintained the classical M1 and M2 nomenclature, considering that it can be translated by M(LPS + IFN-*γ*) and M(IL-4 + IL-13) macrophages, respectively [26].

In this study, RAW 264.7 cells and BMDMs were polarized into M1 macrophages, after 18 hours of stimulation with LPS and IFN-*γ*, while the M2 phenotype was obtained with IL-4 and IL-13, using well-described protocols [22], except for the LPS concentration, which was reduced to 10 ng/mL to limit cytotoxicity (data not shown). IFN-*γ* alone, compared to the LPS + IFN-*γ* cocktail, was unable to polarize RAW 264.7 cells into M1 cells, with TNF-*α* being the only M1 marker gene slightly induced, with increased production of secreted TNF-*α*, which is in agreement with the data of our group on THP-1 derived macrophages [54].

Polarization was assessed by monitoring the expression of several specific marker genes mainly at the mRNA level and also at the protein level for some markers.* iNOS*,* IL-6*,* Arg2*,* TNF-α*, and IL-12 were chosen as proinflammatory M1 markers,* Arg1*,* MRC1*,* Mgl2*,* YM1*, and IL-10 were chosen as M2 markers, and* HO-1* and* Srxn1* were chosen as MOX and redox-sensitive markers [30–33], although we are aware that, whatever the model used, M1 and M2 cells represent two extremes of a spectrum of macrophage functions.

Our results in RAW 264.7 macrophages were consistent with data obtained with murine bone marrow-derived macrophages from the study of Khallou-Laschet et al. in 2010 [30] and with BMDMs characterized in this study (Figure 2). Indeed, as Khallou-Laschet et al., we observed an overexpression of not only* iNOS* and* IL-6* but also* Arg2*,* TNF-α*, and IL-12 in M1 macrophages. M2 cells overexpressed* Arg1* and* MRC1* as well as* Mgl2, YM1*, and IL-10, often considered as canonical M2 markers, at least in mice (for* Arg1*,* Mgl2*, and* YM1*) [30, 55].

In addition to avoiding bone marrow or elicited peritoneal macrophage isolation from mice and being useful and convenient in studies related to atherosclerosis, the RAW 264.7 polarized macrophages could also be used in the context of other chronic inflammatory diseases including cancer, particularly to dissect signaling mechanisms or design coculture models.

Once this model of polarized macrophages was validated, we investigated the effects of native LDLs or oxidized LDLs on macrophage polarization, since LDLs play a major role in atherogenesis and in particular oxidized LDLs [56, 57]. The latter accumulating in the lesion could contribute to changes into the macrophage microenvironment modulating their phenotypes and functions. Within the lesion, oxidized LDLs are recognized by scavenger receptors (e.g., CD36, SR-A1, and LOX-1), mainly expressed by macrophages, leading to the internalization of modified LDLs. Scavenger receptors, contrary to the native LDL receptors, are not regulated by intracellular cholesterol, contributing to unregulated lipid loading, leading to foam cells [5, 58]. But how oxidized LDLs interfere with macrophage polarization remains largely unexplored. By the way, most of the available data is focused on copper-oxidized LDLs, oxidized at both the protein and lipid moieties and particularly enriched in lipid hydroperoxides. Hirose et al. (2011) evaluated the effects of Ox-LDLs on human polarized macrophages, using microarrays, but after 6 hours of incubation, which clearly affects the expression profiles differentially in M0, M1, and M2 cells but remains short for assessing polarizing effects, requiring periods of time of at least 18 hours. It has to be mentioned that only copper-oxidized LDLs were evaluated and gene expression was assessed exclusively at the mRNA level [32]. Isa et al. (2011) also assessed the impact of Ox-LDLs on human polarized macrophages. They determined that M2 macrophages are more sensitive to the lipotoxicity induced by Ox-LDLs, as compared to M0 macrophages and to monocytes. Anew, in the latter study, only copper-oxidized LDLs were evaluated and only M0 and M2 macrophages were taken into account [59]. van Tits et al. (2011) have shown that Ox-LDLs enhance proinflammatory responses stimulated by LPS in human M2 macrophages, which is in agreement with our data showing proinflammatory effects of Ox-LDLs. They hypothesized that this phenotypic switch could play a role in atherogenesis, but, again in this study, only copper-oxidized LDLs were used [51].

However, other oxidized forms of LDLs, probably more relevant, have been described, such as myeloperoxidase-oxidized LDLs [60, 61]. The latter display specific modifications of Apo-B100 and their lipid hydroperoxide content remains low [36]. Calay et al. already showed that Ox-LDLs and MpOx-LDLs induce different responses in RAW 264.7 unpolarized macrophages, with MpOx-LDLs being the most potent in activating Nrf2 and in lipid loading [8]. That is why we wanted to pursue this comparative study on Ox-LDLs and MpOx-LDLs but taking into account the macrophage phenotype.

In this study, we first wondered whether oxidized LDLs could by themselves polarize macrophages, given the paucity of available data. From our data, we can conclude that oxidized LDLs do not lead to a defined polarized phenotype. However, the expression of several marker genes is affected in particular by MpOx-LDLs which favor the expression of not only both redox-sensitive and M2 marker genes but also M1 marker genes. Calay et al. have already shown that MpOx-LDLs more efficiently activate Nrf2, at least in M0 cells, but further studies will be required to unravel more in detail the signaling pathways activated by different kinds of oxidized LDLs in macrophages [8, 62]. Our data suggest that qualitative differences in LDL composition in the atheroma could modulate macrophage polarization and play a role in the evolution of the lesion. Khallou-Laschet and coworkers showed that M2 macrophages were predominant in early atherosclerotic lesions of ApoE KO mice, while M1 macrophages were the predominant phenotype in advanced lesions. It remains unclear whether M2 macrophages are progressively replaced by M1 cells or whether there is a phenotypic switch [30]. However, data from Davis recently showed that* in vitro* macrophages could be able to repolarize by themselves in response to changes in the microenvironment (e.g., cytokines) in the context of an infection with* Cryptococcus neoformans *[63]. It will be worthwhile in the future to confirm the phenotypic distribution of macrophages in atheromas taking into account the M1 and M2 cells in lesions using alternative murine models closer to the human situation.

We then evaluated the effects of LDLs on polarized macrophages, focusing on marker gene expression. Regarding M1 cells, we observe no major effect of LDLs, whether oxidized or not, neither on the expression of* iNOS* (mRNA) (except for an increased* iNOS* expression in M1 cells in the presence of MpOx-LDLs) nor on the expression of IL-6 (protein). However, in RAW 264.7 M0 cells, MpOx-LDLs, contrary to Ox-LDLs, seemed to induce an overexpression of* Arg1*,* MRC1* (mRNA), and IL-10 (protein) well described as M2 markers and anti-inflammatory genes [64]. In M1 macrophages,* Arg1* was significantly overexpressed in the presence of MpOx-LDLs. The M2 phenotype is generally maintained (Nat-LDLs and Ox-LDLs) or even reinforced (MpOx-LDLs) regarding* MRC1*,* Arg1*, and* YM1* expression and IL-10 secretion, with some variations between RAW 264.7 cells and BMDMs. Interestingly, oxidized LDLs also favor the expression of the protective antioxidant enzymes HO-1 and Srxn1 not only in M1 cells but also in M0 and M2 cells, in particular for MpOx-LDLs in RAW 264.7 cells. When comparing quantitatively RAW 264.7 macrophages and BMDMs, the response in BMDMs was less pronounced. We cannot exclude the fact that BMDMs would have already initiated M2 polarization due to the presence of M-CSF in the macrophage-differentiation conditioned medium.

Polarized macrophages (RAW 264.7 or BMDMs) also showed an important ability to become foam cells when incubated with oxidized LDLs (Figures 6 and 7). Our data suggest that M1 and M2 macrophages have a high potential to engulf lipids, in particular when they are stimulated with MpOx-LDLs. Surprisingly, in addition to taking up oxidized LDLs, M1 macrophages also engulf native LDLs (Figure 6). This phenomenon could be explained by their likely exacerbated oxidative metabolism activated by LPS. As a consequence, this could lead to oxidation of Nat-LDLs during the 24-hour incubation, allowing their intracellular accumulation [12, 15]. While the cholesterol content increased in BMDMs incubated with LDLs (Figure 7(b)), this could not be demonstrated in RAW 264.7 cells.

Our data show that the number (and size) of lipid droplets is higher in macrophages incubated with MpOx-LDLs whatever the phenotype in both RAW 264.7 cells and BMDMs. Figure 7(b) shows that, in BMDMs, total cholesterol is also comparable in MpOx-LDLs-treated BMDMs whatever the phenotype. These data suggest that the uptake of MpOx-LDLs by itself is not sufficient to explain the changes in gene expression. One possible hypothesis that could explain our data (at least in part) could be the presence of different combinations of miRNAs. Polarized macrophages display different miRNA profiles ([65, 66] both on human monocyte-derived macrophages). Oxidized LDLs also affect miRNA profiles [62]. But, to our knowledge, there are no data comparing miRNA expression profiles in macrophages taking into account both the macrophage phenotype and different LDL oxidation protocols. But one can imagine that some of the anti-inflammatory and antioxidant effects induced by MpOx-LDLs we observe could be explained by different miRNA combinations resulting from the polarization process and varying according to the type of LDLs used (Nat-LDLs, Ox-LDLs, or MpOx-LDLs).

Altogether these data suggest that MpOx-LDLs are rather anti-inflammatory, in particular by favoring the overexpression of* Arg1,* and protective, inducing antioxidant enzymes such as HO-1 and Srxn1, while the effects are less clear for Ox-LDLs. Our data are original, since we could not find any study comparing the effects of Ox-LDLs and MpOx-LDLs on polarized macrophages. LDLs, oxidized or not, did not affect the phagocytosis potential whatever the phenotype in both RAW 264.7 cells and BMDMs.

## 5. Conclusion

In conclusion, an easy-to-set up and easy-to-use model of polarized RAW 264.7 has been described and used in this study to better distinguish the effects of copper-oxidized and myeloperoxidase-oxidized LDLs on polarized macrophages. Our data suggest that MpOx-LDLs are the most effective in modulating marker gene expression in unpolarized cells and in inducing an anti-inflammatory and anti-oxidant phenotype compared to Ox-LDLs and finally seem also to be the most efficiently cleaned up by the macrophages whatever their phenotype, which is in agreement with the data of Calay et al. (2010) [8] on murine M0 cells and the data of van Tits et al. (2011) on human M1 and M2 cells [51]. Further studies will be needed in order to determine the specific roles of those macrophages in atherosclerosis (genesis, progression, and regression), taking into account different forms of more relevant oxidized LDLs.

## Supplementary Material

Supplementary figures support our data showing an overexpression of M2 markers at the mRNA level (Mgl2, YM1, Arg1 and MRC1) (Figures S1 and S5) and at the protein level (IL-10) (Figure S3), when M0, M1 and M2 macrophages are stimulated with MpOx-LDLs. There is no marked effect of MpOx-LDLs on M1 markers at the mRNA level (Arg2 and TNF-*α*) (Figure S2) and at the protein level (IL-12 and TNF-*α*) (Figure S3). Figure S4 shows the effects of LDLs on the secretion of cytokines (IL-10, IL-12 and TNF-*α*) in RAW 264.7 M0 macrophages stimulated with Nat-LDLs, Ox-LDLs or MpOx-LDLs for 24 hours. Figure S6 shows the impact of native or oxidized LDLs on the phagocytosis of fluorescent beads by (un)polarized RAW 264.7 macrophages and BMDMs. Materials and methods are described in the Materials and Methods section of the manuscript.

## Abbreviations

Arg1: Arginase-1
Arg2: Arginase-2
BMDMs: Bone marrow-derived macrophages
CD36: Cluster of differentiation 36
DMPC: 1,2-Dimyristoyl-*sn*-glycero-3-phosphocholine
GM-CSF: Granulocyte-macrophage colony-stimulating factor
HO-1: Heme oxygenase-1
IL-4: Interleukin-4
IL-6: Interleukin-6
IL-10: Interleukin-10
IL-12: Interleukin-12
IL-13: Interleukin-13
iNOS: Inducible Nitric Oxide Synthase
LDL-R: Low-density lipoprotein receptor
LPS: Lipopolysaccharide
M-CSF: Macrophage colony-stimulating factor
Nat-LDLs: Native low-density lipoproteins
M1: Proinflammatory macrophages or “classically” activated macrophages
M2: Anti-inflammatory macrophages or “alternatively” activated macrophages
MCP-1: Monocyte chemoattractant protein-1
Mgl2: Macrophage galactose N-acetyl-galactosamine specific lectin 2
MOX: Oxidized phospholipid-induced macrophages
MpOx-LDLs: Myeloperoxidase-oxidized low-density lipoproteins
MPO: Myeloperoxidase
MRC1: Mannose receptor type-C1
Nrf2: Nuclear factor erythroid 2-related factor 2
Ox-LDLs: Copper sulfate-oxidized low-density lipoproteins
OxPAPC: Oxidized 1-palmitoyl-2-arachidonoyl-sn-glycero-3-phosphorylcholine
PFA: Paraformaldehyde
ROS: Reactive oxygen species
RNS: Reactive nitrogen species
SR-A1: Scavenger receptor type A1
Srxn1: Sulfiredoxin-1
TNF-*α*: Tumor necrosis factor-*α*
WHO: World Health Organization
YM1: Beta-N-acetylhexosaminidase, also known as Chil3, chitinase-like protein 3.
