# Supplementary material for: Myeloperoxidase-Oxidized LDLs Enhance an Anti-Inflammatory M2 and Antioxidant Phenotype in Murine Macrophages
Source: Mediators Inflamm. 2016 Aug 30;2016:8249476. doi: 10.1155/2016/8249476 (PMC5021486; doi:10.1155/2016/8249476)
Supplement: Supplementary file 1 — Supplementary figures support our data showing an overexpression of M2 markers at the mRNA level (Mgl2, YM1, Arg1 and MRC1) (Figures S1 and S5) and at the protein level (IL-10) (Figure S3), when M0, M1 and M2 macrophages are stimulated with MpOx-LDLs. There is no marked effect of MpOx-LDLs on M1 markers at the mRNA level (Arg2 and TNF-α) (Figure S2) and at the protein level (IL-12 and TNF-α) (Figure S3). Figure S4 shows the effects of LDLs on the secretion of cytokines (IL-10, IL-12 and TNF-α) in RAW 264.7 M0 macrophages stimulated with Nat-LDLs, Ox-LDLs or MpOx-LDLs for 24 hours. Figure S6 shows the impact of native or oxidized LDLs on the phagocytosis of fluorescent beads by (un)polarized RAW 264.7 macrophages and BMDMs. Materials and methods are described in the Materials and Methods section of the manuscript. [file 8249476.f1.pdf]

Supplementary data

Figure S1

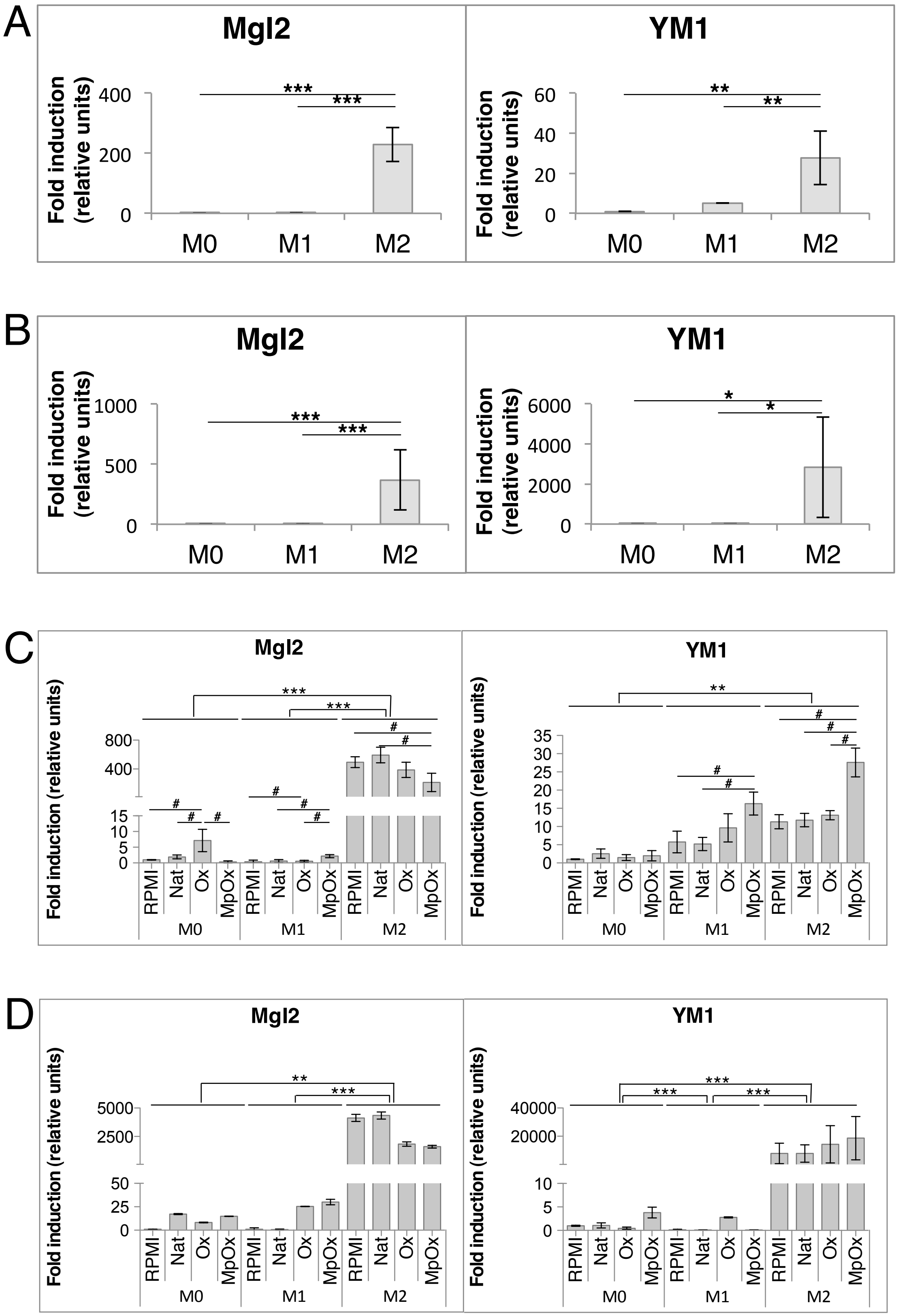

Supplementary data

Figure S2

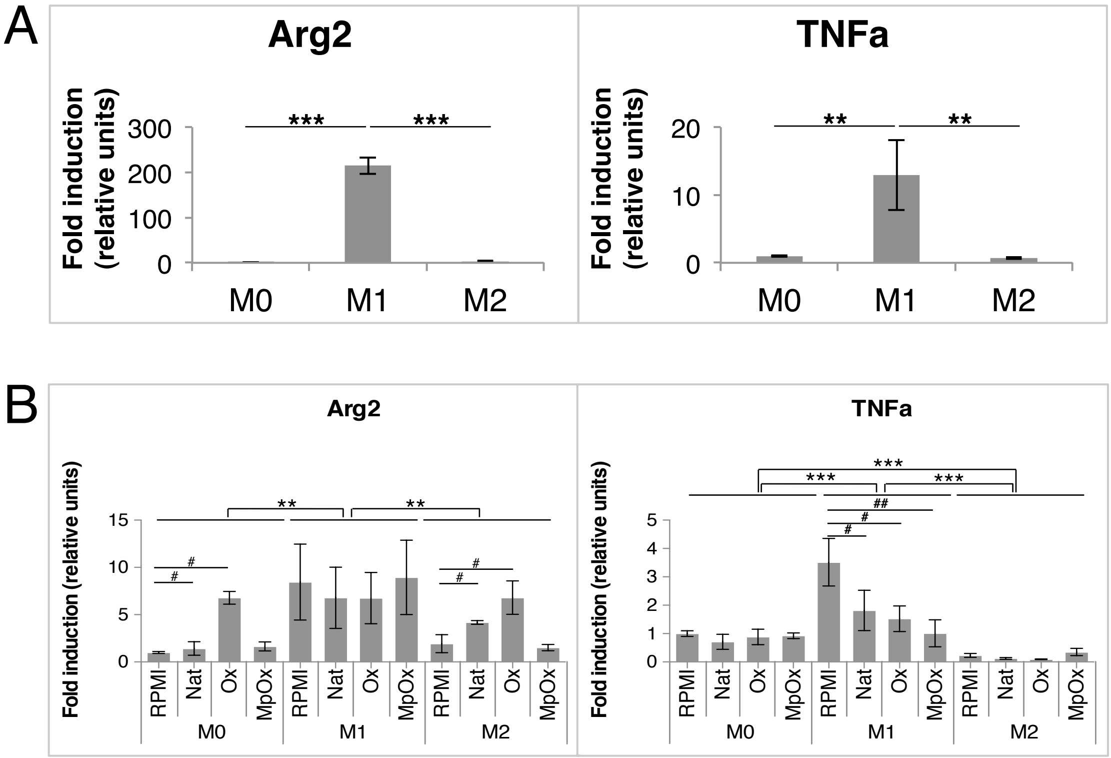

Supplementary data

Figure S3

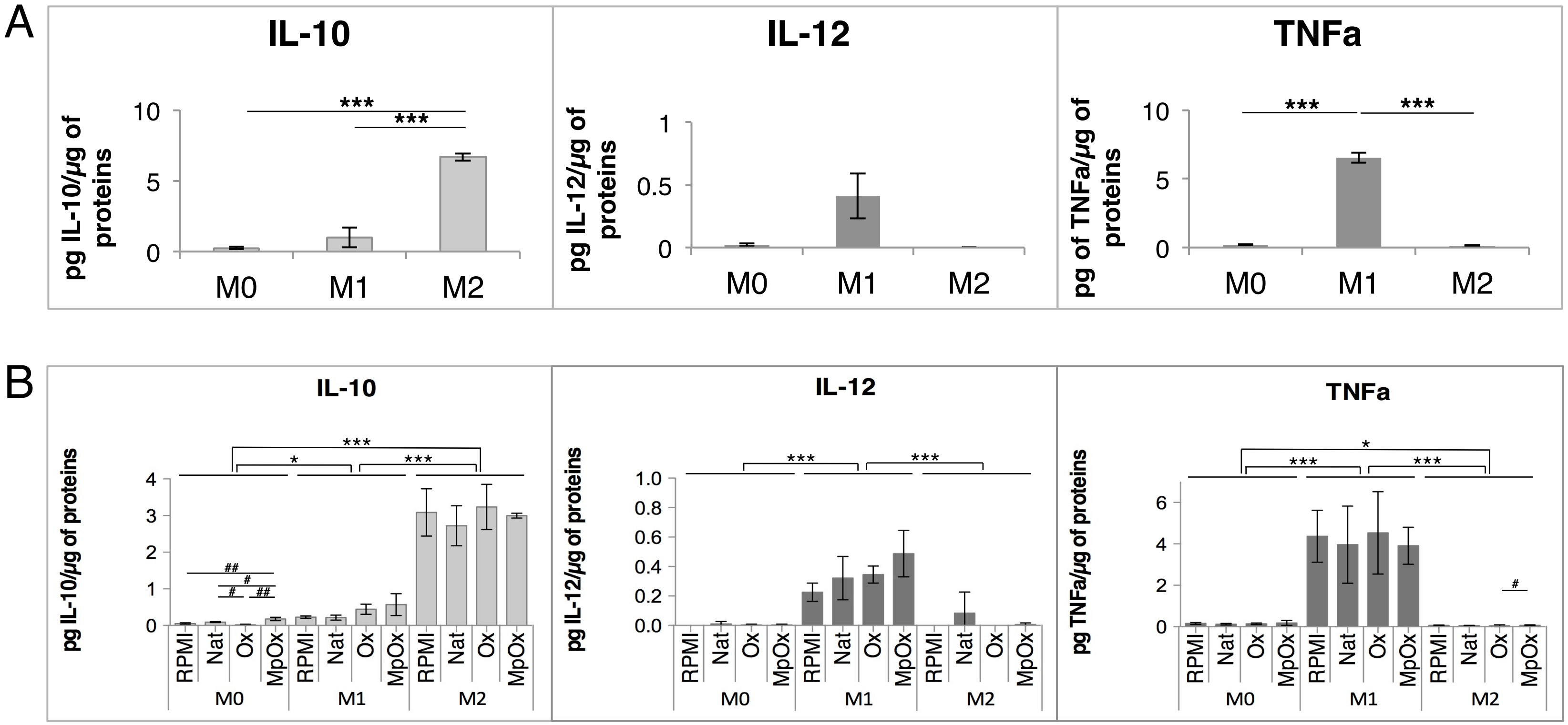

Supplementary data

Figure S4

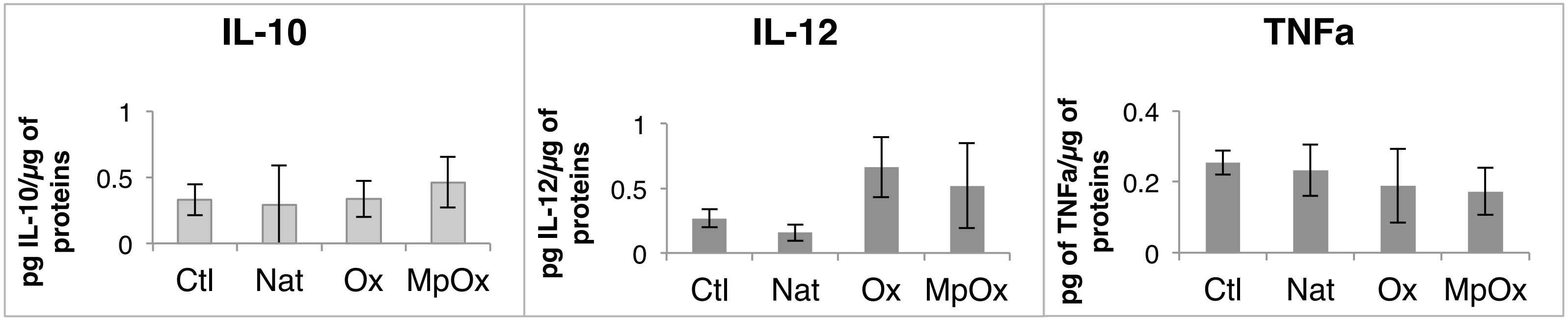

Supplementary data

Figure S5

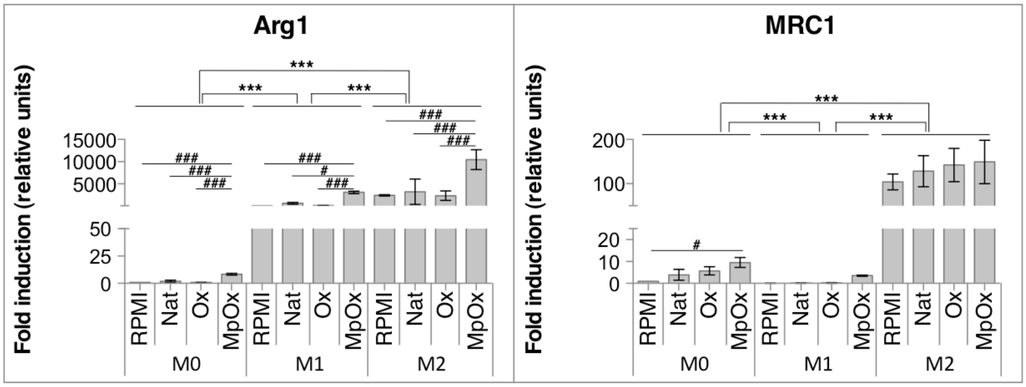

Supplementary data

Figure S6

A

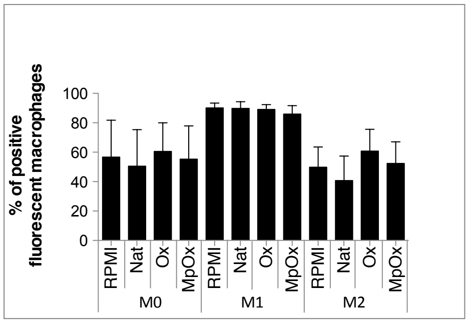

B

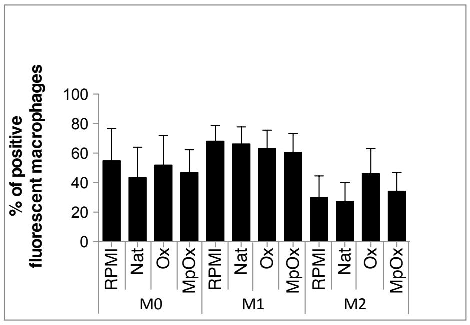

Supplementary data

Supplementary Figure Legends

Figure S1. Expression of M2 polarization marker genes (*Mgl2* and *YM1*) at the mRNA level (RT-qPCR), in M0, M1 and M2 macrophages, in the absence (A-B) or in the presence of LDLs for 24 hours (100 µg/ml) (C-D), in RAW 264.7 cells (A and C) and in BMDMs (B and D). (A-B) The expression of the M2 marker genes was analyzed by RT-qPCR as described in figure 1. Data is expressed as mean fold induction relatively to M0 cells ± SD (n = 6 in RAW 264.7 (A); n = 5 in BMDMs (B)). ANOVA 1: * p < 0.05; ** p < 0.01; *** p < 0.001.

(C-D) Comparative effects of LDLs on (un)polarized RAW 264.7 macrophages and BMDMs. M0, M1 and M2 macrophages were stimulated in the presence or not (RPMI control) of Nat-LDLs, Ox-LDLs, MpOx-LDLs for 24 hours (100 µg/ml).The expression of polarization marker genes was assessed at the mRNA level (RT-qPCR) in RAW 264.7 cells (C) and BMDMs (D). Data for *Mgl2* and *YM1* was analyzed by a Kruskal-Wallis ANOVA on ranks. Data was normalized with TBP used as housekeeping gene and expressed as mean fold induction relatively to M0 control cells (RPMI) ± SD (n = 6 in RAW 264.7 (C); n = 5 in BMDMs (D)). ANOVA 2: *#, p < 0.05; **, ## p < 0.01; ***, ### p < 0.001.

Figure S2. Expression of M1 polarization marker genes (*Arg2* and *TNFα*) at the mRNA level (RT-qPCR), in M0, M1 and M2 macrophages, in the absence (A) or in the presence of LDLs for 24 hours (100 µg/ml) (B) in RAW 264.7 cells. (A) The expression of M1 polarization marker genes was assessed at the mRNA level (RT-qPCR). Data is expressed as mean fold induction relatively to M0 cells ± SD (n = 6). ANOVA 1: * p < 0.05; ** p < 0.01; *** p < 0.001.

(B) Comparative effects of LDLs on (un)polarized RAW 264.7 macrophages. M0, M1 and M2 macrophages were stimulated in the presence or not (RPMI control) of Nat-LDLs, Ox-LDLs, MpOx-LDLs for 24 hours (100 µg/ml). Data for *Arg2* and *TNFα* was analyzed by a Kruskal-Wallis ANOVA on ranks. Data was normalized with TBP used as housekeeping gene and expressed as mean fold induction relatively to M0 control cells (RPMI) ± SD (n = 6). ANOVA 2: *, # p < 0.05; **, ## p < 0.01; ***, ### p < 0.001.

Figure S3. Secretion of cytokines by RAW 264.7 M0, M1 and M2 polarized macrophages in the absence (A) or in the presence of LDLs for 24 hours (100 µg/ml) (B). (A) Secreted IL-10 (M2 marker) as well as IL-12 and TNFα (M1 markers) were assessed by ELISA. (B) Comparative effects of LDLs on (un)polarized RAW 264.7 macrophages. M0, M1, and M2 macrophages were stimulated in the presence or not (RPMI control) of Nat-LDLs, Ox-LDLs, MpOx-LDLs for 24 hours (100 µg/ml). IL-10, IL-12 and TNFα cytokines were assessed in the cell culture supernatants by ELISA. Data is expressed relatively per µg protein per well as mean ± SD (n = 3). (A) ANOVA 1, (B) ANOVA 2: *, # $p < 0.05$; **, ## $p < 0.01$; ***, ### $p < 0.001$.

Figure S4. Comparative effects of LDLs on the secretion of M1 (IL-12 and TNFα) and M2 (IL-10) cytokines by unpolarized RAW 264.7 M0 macrophages. M0 macrophages were treated for 24 hours in the presence of medium alone (Ctl), Native-LDLs (Nat), Ox-LDLs (Ox) or MpOx-LDLs (MpOx) (100 µg/ml). Cytokines were assessed in the supernatants by ELISA. Data is expressed relatively per µg protein per well as mean ± SD (n = 3). ANOVA 1: * $p < 0.05$; ** $p < 0.01$; *** $p < 0.001$.

Figure S5. Comparative effects of LDLs on the expression of M2 marker genes (*Arg1* and *MRC1*) in RAW 264.7 (un)polarized macrophages. Zoomed data for *Arg1* and *MRC1* from Fig. 4A.

Macrophages were treated for 24 hours in the presence of medium alone (RPMI control), Native-LDLs (Nat), Ox-LDLs (Ox) or MpOx-LDLs (MpOx) (100 µg/ml) and the expression of polarization marker genes was monitored at the mRNA level (RT-qPCR), with *Arg1* and *MRC1* as M2 markers. Data was normalized with TBP used as housekeeping gene and expressed as mean fold induction relatively to M0 control cells (RPMI) ± SD (n = 6). ANOVA 2: * $p < 0.05$; ** $p < 0.01$; *** $p < 0.001$.

Figure S6. Impact of native or oxidized LDLs on the phagocytosis of fluorescent beads by (un)polarized RAW264.7 cells (A) and BMDMs (B). M0, M1 and M2 macrophages were incubated in the presence or not of Nat-, Ox- and MpOx-LDLs and fluorescent beads (dilution: 1/133) for 24 hours. The percentage of positive fluorescent cells was evaluated using flow cytometry (FACS BD Verse). Data is expressed as mean ± SD ($n = 3$). ANOVA 2: *, # $p < 0.05$; **, ## $p < 0.01$; ***, ### $p < 0.001$.
